# The Effectiveness of an Artificial Intelligence–Based Gamified Intervention for Improving Maternal Health Outcomes Among Refugees and Underserved Women in Lebanon: Community Interventional Trial

**DOI:** 10.2196/65599

**Published:** 2025-11-04

**Authors:** Shadi Saleh, Nour El Arnaout, Nadine Sabra, Asmaa El Dakdouki, Zahraa Chamseddine, Randa Hamadeh, Abed Shanaa, Mohamad Alameddine

**Affiliations:** 1 Global Health Institute American University of Beirut Beirut Lebanon; 2 Department of Health Management and Policy Faculty of Health Sciences American University of Beirut Beirut Lebanon; 3 Ministry of Public Health Beirut Lebanon; 4 United Nations Relief and Works Agency for Palestine Refugees in the Near East Beirut Lebanon; 5 College of Health Sciences University of Sharjah Sharjah United Arab Emirates

**Keywords:** maternal health, gamification, AI, artificial intelligence, mHealth, mobile health, neonatal outcomes, ANC, antenatal care, antenatal, maternal, mother, game, refugees, refugee, woman, Lebanon, neonatal, pregnant women, pregnancy, pregnant, effectiveness, Lebanese, mHealth tools, medical history, regression model

## Abstract

**Background:**

In Lebanon, disadvantaged pregnant women show poor maternal outcomes due to limited access to antenatal care (ANC) and a strained health care system, compounded by ongoing conflicts and a significant refugee population. Despite substantial efforts to improve maternal health, the provision of maternal health services in primary health care centers (PHCs) still faces significant challenges. Mobile health (mHealth) interventions, particularly those using artificial intelligence (AI) and gamification, are proving effective in addressing gaps in maternal health services by offering scalable and accessible care.

**Objective:**

This study aimed to evaluate the effects of an AI-based gamified intervention, Gamification and Artificial Intelligence and mHealth Network for Maternal Health Improvement (GAIN MHI), on maternal health outcomes and uptake of ANC services among disadvantaged populations in Lebanon.

**Methods:**

The study was a community interventional trial with historical controls, conducted across 19 randomly allocated PHCs in 5 Lebanese governorates. Participants included pregnant women in their first trimester visiting PHCs. The intervention used mHealth tools, including educational mobile-based messages, appointment reminders, and the GAIN MHI app, which provided AI-driven and gamified learning for health care providers (HCPs). Data collected covered demographics, medical history, and maternal and neonatal health outcomes. Key outcome measures included uptake of health care services (eg, ANC visits, supplement intake, ultrasound completion, lab tests) and maternal and neonatal outcomes (eg, term delivery, normal delivery, abortion rate, neonatal morbidity, maternal complications).

**Results:**

This study included 3989 participants, divided between a control group (n=1993, 50%) and an intervention group (n=1996, 50%). Regression models adjusting for demographics, health, and obstetric characteristics showed significantly higher odds in the intervention group for completing 4 or more ANC visits (odds ratio [OR] 1.569, 95% CI 1.329-1.852, *P*<.05), completing lab tests (OR 1.821, 95% CI 1.514-2.191, *P*<.05), 2 or more ultrasound screenings (OR 7.984, 95% CI 6.687-9.523, *P*<.05), urine analysis (OR 4.399, 95% CI 3.631-5.330, *P*<.05), and supplement intake (OR 3.508, 95% CI 2.982-4.128, *P*<.05). Regarding outcomes, the intervention group had 29.5% increased odds of a term delivery (OR 1.295, 95% CI 1.095-1.532, *P*=.002) and 58% increased odds of avoiding neonatal morbidity (OR 1.580, 95% CI 1.185-2.108, *P*=.002). However, both groups showed decreased odds of normal delivery (intervention: OR 0.774, 95% CI 0.657-0.911; control: OR 0.823, 95% CI 0.701-0.964) and increased odds of maternal complications (intervention: OR 0.535, 95% CI 0.449-0.637; control: OR 0.586, 95% CI 0.474-0.723; *P*<.05).

**Conclusions:**

The GAIN MHI intervention effectively improves uptake of ANC and maternal and neonatal outcomes. Our findings highlight the potential of mHealth interventions to enhance health care delivery. To sustain these improvements, future research should focus on integrating mHealth with other interventions that address socioeconomic and contextual factors. This approach will further optimize maternal and neonatal health outcomes among disadvantaged populations.

## Introduction

### Overview of Maternal Health in Lebanon

Women and girls, especially in conflict-affected and displaced communities, are highly vulnerable to poor sexual, reproductive, and maternal health (SRMH) [1-4]. Disadvantaged pregnant women in Lebanon encounter various maternal health complications and adverse pregnancy outcomes, including higher-than-usual cesarean section (C-section) deliveries due to pregnancy complications, preterm births, and hemorrhages, among others [5-7]. One key challenge is the limited access to antenatal care (ANC) services [8-10]. Recent investigations have underscored a remarkable decline in the use of ANC services among disadvantaged pregnant women in Lebanon, falling below the recommended threshold of 4 visits [1,6,11,12]. Barriers to accessing ANC include transportation issues, distance to primary health care centers (PHCs), shortage of skilled health care providers (HCPs), lack of awareness about available services, and the financial burden associated with seeking care [6,11,13-15]. Addressing these challenges and ensuring equitable access to maternal health care services are essential steps to improving pregnancy outcomes and reducing maternal mortality rates [16,17].

### The Refugee Crisis in the Context of Lebanon

Addressing the identified barriers to accessing ANC services becomes particularly challenging in a fragile context, such as Lebanon, the country hosting the highest number of refugees worldwide. Around 1.5 million Syrian refugees, 489,292 Palestinian refugees, and 11,645 refugees from various other nationalities reside in Lebanon [18-20]. The continuous influx of refugees has strained the Lebanese health care system, resulting in medication shortages and high health care costs [21-25]. The country also faces a significant migration of health care professionals due to the protracted economic crisis it has witnessed over the past few years [22,26]. As a result, accessing basic health and social services has become particularly challenging for host communities, further exacerbating their vulnerability [27]. Among refugees, women of childbearing age face particular health care challenges, highlighting the importance of addressing women’s health issues in Lebanon [28,29].

### Primary Health Care Service Use Among Pregnant Women in Lebanon

To access essential medical services in Lebanon, disadvantaged Lebanese populations and Syrian refugees often resort to a network of PHCs, affiliated with the Ministry of Public Health (MOPH) [30,31]. The MOPH has established around 200 PHCs, managed by nongovernmental organizations (NGOs), municipalities, and governmental institutions and distributed across the country [32,33]. These PHCs provide affordable health care services for over 1 million disadvantaged Lebanese populations and Syrian refugees annually, with Lebanese patients benefiting from reduced out-of-pocket fees and Syrian refugees being supported by subsidies from the United Nations High Commissioner for Refugees (UNHCR) [31,34]. Alternatively, Palestinian refugees access health care services via a channel of 27 PHCs administered by the United Nations Relief and Works Agency for Palestine Refugees in the Near East (UNRWA) [30]. Both the MOPH and the UNRWA provide services related to reproductive health, including family planning, ANC, and postnatal care (PNC) services [35-37]. Recognizing the strains on Lebanon’s health care system due to the influx of refugees and in alignment with its commitment to ensure universal health coverage, the MOPH collaborates with partners, such as the World Bank, the United Nations Populations Funds (UNFPA), and the European Union (EU), to implement projects aimed at strengthening primary health care services, including reproductive health care [23,31,32,38-41]. These initiatives provide wellness and sexual reproductive health (SRH) packages covering services such as screening, consultation, prescription medications, ANC, and PNC [39,40,42]. Similarly, to sustain its health care initiatives, the UNRWA relies on funding from member states of the United Nations, the EU, and various other states. Yet, despite the availability of these subsidized maternal health care services, compliance with standards continues to be a serious concern among disadvantaged populations in Lebanon [1]. To address the suboptimal maternal health services, mobile health (mHealth) interventions emerge as a potential solution to improve access to essential health care services [43].

### Role of mHealth Interventions in Enhancing Maternal Health Outcomes

The proliferated usage of mobile devices has catalyzed the widespread adoption of mHealth initiatives, revolutionizing the landscape of health care delivery and empowering individuals to actively engage in enhancing their well-being through accessible and innovative digital platforms [43-47]. mHealth emerges as a critical component for the worldwide effort to decrease maternal mortality and improve maternal health outcomes, mainly within disadvantaged populations [48,49]. By leveraging mobile technology, mHealth interventions offer scalable solutions to address maternal health challenges, providing vital resources, education, and support to pregnant women and new mothers, in remote or underserved areas [43]. mHealth interventions, such as Mobile Technology for Community Health (MOTECH), MOM Connect, and Mobile Alliance for Maternal Action (MAMA), highlight the effectiveness of strategies such as sending voice note messages and appointment reminders to expectant mothers and their spouses [50-52]. These interventions not only facilitate timely access to essential maternal health services but also promote greater engagement and support throughout the prenatal and postnatal journey. The integration of mHealth interventions extends beyond empowering the pregnant women and their spouses, by targeting their HCPs as well. Initiatives such as the Safe Delivery Application (SDA) show that such interventions have positively impacted HCPs’ knowledge and skills, resulting in tangible improvements in maternal and neonatal outcomes.

As mHealth continues to advance, artificial intelligence (AI) innovations are being increasingly integrated into the health care sector to enhance the quality of care and address various health areas [53,54]. AI has been used in diagnostic support, as well as to enhance access to virtual health services in remote areas and address knowledge and skill gaps [55]. Building on these advancements, AI has shown significant promise in maternal health by predicting complications, such as preeclampsia, postpartum depression, gestational diabetes and anemia, stillbirths, and preterm deliveries [56]. Additionally, AI shortens the training time required for health care professionals in assessing preterm deliveries and improves access to maternal health care [57,58].

Moreover, the concept of gamification has been incorporated into mobile platforms to train HCPs and inform individuals about health and illness management [59-62]. For instance, the Maternal and Newborn Technology for Resilience in Rural Areas (MANTRA) app applies gamification to increase awareness regarding maternal and child health risk factors [63]. Users expressed satisfaction with the gamification approach, which led to improved maternal and child health knowledge and more informed decision-making [63].

These findings underscore the profound efficacy of mHealth interventions in maternal health outcomes and the accessibility of maternal health services [64,65].

### mHealth Interventions for Maternal Health in Lebanon

In Lebanon, mHealth technologies have been incorporated to improve primary health care services for disadvantaged Lebanese and refugee populations [64,66]. These initiatives focus on using mHealth tools to manage noncommunicable diseases (NCDs) through interventions such as educational SMS messages and appointment reminders [67,68]. Other studies in Lebanon have investigated mHealth for weight management and the uptake of routine mammography [69,70]. In the realm of maternal health, the UNRWA electronic mother and child health (e-MCH) app represents a notable intervention. It integrates a comprehensive database on maternal and infant care and provides appointment reminders, thereby playing a critical role in ensuring the continuity of essential health care services [71].

Although certain countries in the Middle East and North Africa (MENA) region have effectively used mHealth solutions to enhance maternal health literacy and ANC visits, the focus on maternal health in Lebanon remains limited [72]. There is a scarcity of AI-driven solutions or gamified elements, highlighting a critical gap in leveraging technology to improve maternal health outcomes [73].

### Study Aim

This study aimed to assess the effects of an AI-based gamified intervention, Gamification and Artificial Intelligence and mHealth Network for Maternal Health Improvement (GAIN MHI), on maternal health outcomes and uptake of ANC services among disadvantaged populations in Lebanon.

## Methods

### Study Design

This study was a community interventional trial with historical controls at distinct PHCs. Pre- and postintervention data were collected with the help of the assigned research assistant at each center. Data collected included demographics, medical and obstetric history, and maternal and neonatal health outcomes, including neonatal morbidities and maternal complications. The study comprised 3 distinct phases: preintervention data collection, intervention, and subsequent postintervention data collection. The preintervention phase spanned from July to December 2021. The intervention phase took place from September 2021 to December 2022. Finally, the postintervention phase extended from March to June 2023.

### Center Selection

In total, 20 PHCs across 5 governorates in Lebanon—Beirut, Bekaa, North Lebanon, Mount Lebanon, and South Lebanon—were selected for this study. These centers serve disadvantaged Lebanese populations and refugees. Due to logistical constraints, 1 PHC was excluded, resulting in 19 (95%) centers. Of these, 10 (53%) were designated as control centers and 9 (47%) as intervention centers. Among the selected PHCs, 9 (47%; both control and intervention) are affiliated with the MOPH and serve Syrian refugees and disadvantaged Lebanese populations. The remaining 10 (53%) PHCs operate under the UNRWA and serve Palestinian refugees in Lebanon.

### Study Population

Our target population in both pre- and postintervention phases consisted of pregnant women who presented at the selected PHCs during their first trimester and continued visiting the health centers for at least a year. All age groups were included. In the preintervention phase, pregnant women attended 1 of the PHCs for ANC during their first trimester between August 2018 and August 2020 and continued visiting the center until their delivery or termination of the pregnancy. However, in the postintervention phase, pregnant women were already enrolled in the intervention and visited the PHCs during the period of the intervention. Controls were collected using the historical medical records of the patients at both timepoints.

### The GAIN MHI Intervention

**Figure 1:**
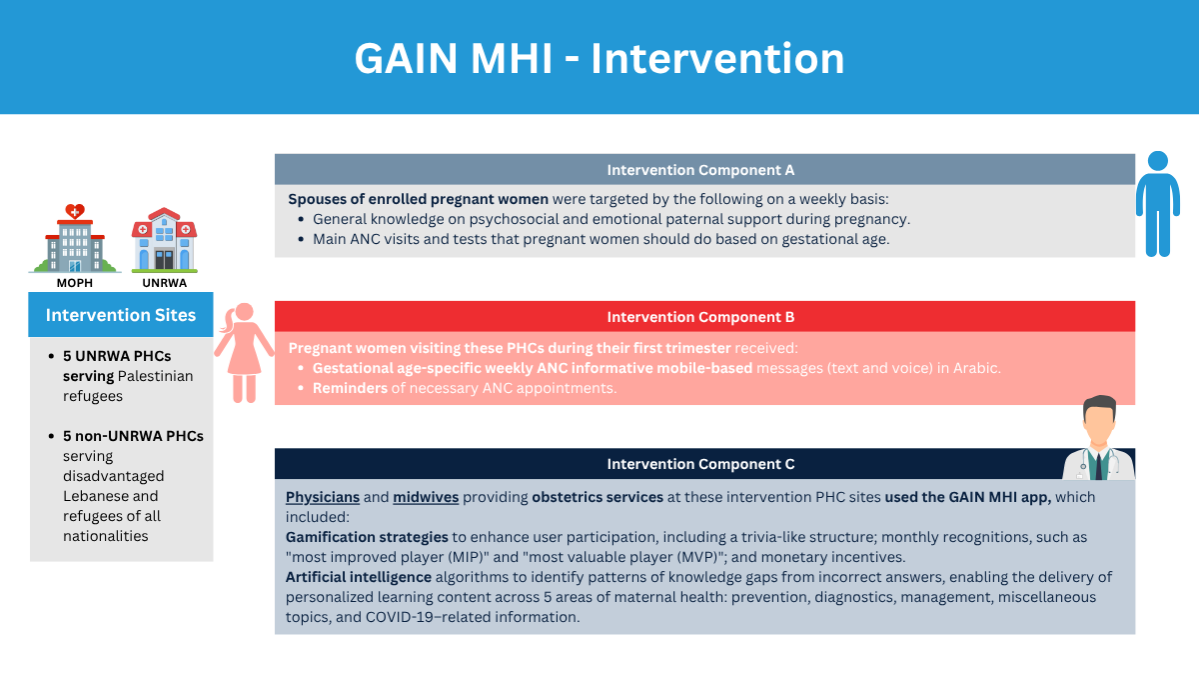
Visual representation of the different components of the GAIN MHI intervention. ANC: antenatal care; GAIN MHI: Gamification, Artificial Intelligence and mHealth Network for Maternal Health Improvement; MOPH: Ministry of Public Health; PHC: primary health care center; UNRWA: United Nations Relief and Works Agency for Palestine Refugees in the Near East.

**Figure 2:**
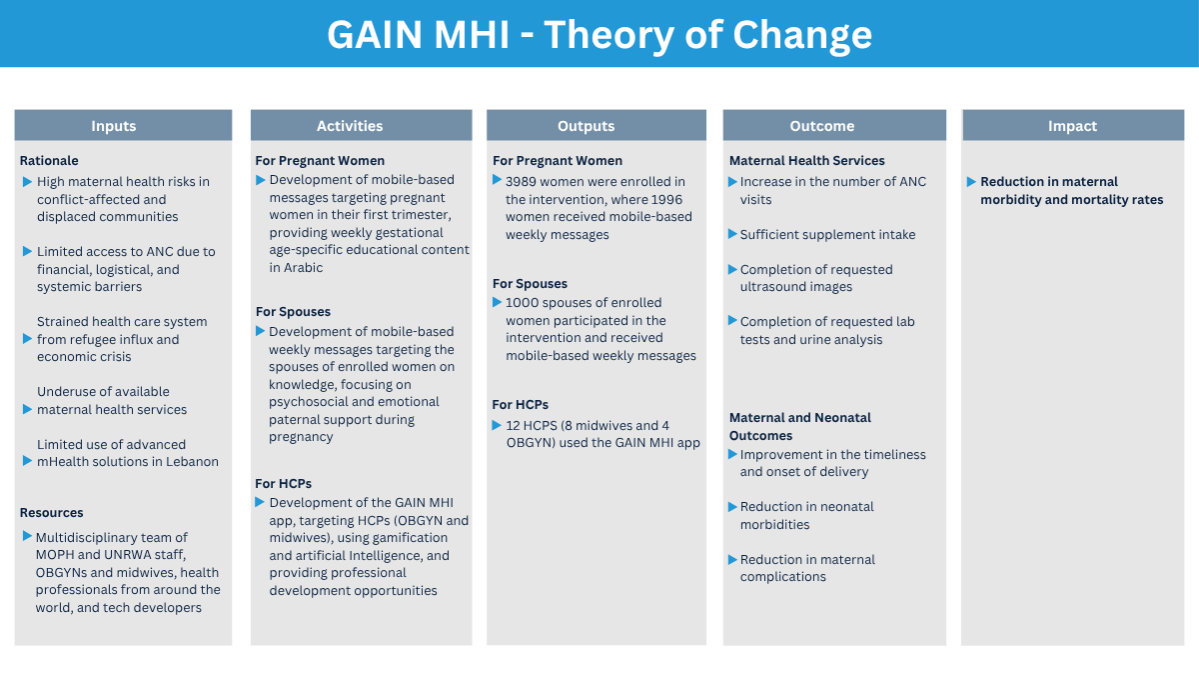
GAIN MHI theory of change. ANC: antenatal care; GAIN MHI: Gamification, Artificial Intelligence and mHealth Network for Maternal Health Improvement; HCP: health care provider; mHealth: mobile health; MOPH: Ministry of Public Health; OBGYN: obstetricians and gynecologists; UNRWA: United Nations Relief and Works Agency for Palestine Refugees in the Near East.

The GAIN MHI intervention (Figure 1) consisted of 2 different modalities: mobile-based messages (text and voice) and a mobile app (GAIN MHI app) aimed at enhancing maternal health outcomes (Figure 2).

The mobile-based messages targeted pregnant women in their first trimester, including disadvantaged Lebanese populations and Syrian and Palestinian refugees, providing weekly gestational age-specific educational content in Arabic. The content covered pregnancy education, nutritional recommendations and supplementation, pregnancy danger signs, and postpartum care for 6 months after delivery, encouraging postpartum visits, breastfeeding, contraception, maternal mental health, and infant vaccinations. Reminder messages were also sent for upcoming ANC visits, vaccinations, tests, and medical examinations. Additionally, weekly messages were sent to the spouses of enrolled women on knowledge, focusing on psychosocial and emotional paternal support during pregnancy.

Although the focus was primarily on supporting pregnant women and their spouses, targeting HCPs was crucial. HCPs face various challenges, such as insufficient continuing education programs and career advancement, which hinder their work, notably in Lebanon [74,75]. To address these gaps, the GAIN MHI app, developed by the Global Health Institute at the American University of Beirut (AUB GHI), integrated gamification and AI concepts for the professional development of HCPs, particularly obstetricians and gynecologists (OBGYN) and midwives, working at the selected PHCs. This app features game principles such as a trivia-like concept, monthly recognition (most improved player [MIP] and most valuable player [MVP]), and monetary incentives to enhance learning engagement. These elements foster a sense of competition, motivation, and continuous improvement among HCPs. Additionally, the AI algorithms used in the app identify knowledge gaps based on the HCPs’ incorrect answers, tailoring subsequent questions to individualize learning across 5 categories related to maternal health: prevention, diagnostic, management, miscellaneous, and COVID-19–related questions. For this purpose, a database of information on prenatal care was created. Topic modeling was used on this database to categorize the different topics of the stored content. Likewise, topic modeling was applied to the question bank, enabling the identification of topics linked to the incorrectly answered questions. Relevant information related to the identified topics was then retrieved and provided to the user. By leveraging AI, the GAINMHI app transforms traditional professional development into individualized efficient processes, minimizing the burden of standardized training programs that may not account for individual variability. In addition to this personalized learning approach, a randomized spin wheel is used for category selection, with each spin providing a question followed by explanations after the user answered. The app also includes a Knowledge Resource Center (KRC), and it communicates with users via automated WhatsApp messages, providing feedback on scores, answers, remaining attempts, monthly performance, and rewards. Available in both English and Arabic, the GAIN MHI app represents an innovative approach to advancing HCP education and maternal care.

Although the professional development of HCPs was an integral part of the intervention, it was not the primary focus of this research and is discussed in more detail in a separate paper.

### Data Collection

In both pre- and postintervention phases, secondary data were collected from the medical charts of pregnant women who presented at the selected PHCs during their first trimester and continued visiting the centers for at least a year. In case a woman got pregnant more than once during the selected study period, her latest pregnancy charts were collected if they met the inclusion criteria of the project, while data on previous pregnancies were excluded. The data extracted from the medical charts of eligible pregnant women covered the following aspects: demographics, medical and surgical history, pregnancy history (supplements and ANC vaccinations taken, the first lab tests and ultrasound scans undergone, and any maternal complications encountered), neonatal complications, and breastfeeding history. Primary data on the knowledge, attitude, and practices (KAP) of women were collected before the intervention through quantitative surveys. The results of the KAP surveys will be discussed in a different paper. Primary data were also collected from pregnant women and their spouses participating in the intervention concerning their satisfaction with the mobile-based messages they were receiving. These data were collected through quantitative surveys at the end of the intervention; the results of these surveys are discussed in another paper. Satisfaction of HCPs with the GAIN MHI app was studied and captured through mixed methods surveys administered at the end of the intervention; the results of these surveys are discussed in a separate paper.

### Ethical Considerations

Ethical approval was obtained from the Institutional Review Board (IRB) of the American University of Beirut (protocol number SBS-2020-0317) and the respective Ethical Committees at the MOPH and the UNRWA. Informed consent was obtained from all individuals participating in the intervention. Participants were informed of their right to opt out of the intervention at any point in time. The IRB approval covers secondary analysis without additional consent. All data were anonymized and deidentified. No compensation was received by participants.

### Study Parameters

Study parameters were extracted and recorded for all pregnant women in the pre- and postintervention phases in all the centers. Table 1 shows the set of indicators that were selected to monitor the effectiveness of the intervention, along with their definitions. The selected parameters were categorized into 2 domains: uptake to health care and maternal and neonatal outcomes. Uptake of health care services included completing more than 4 ANC visits, having at least 2 ultrasound screenings taken, undergoing the available blood tests at the PHCs, undergoing the urine analysis test, and taking supplements during pregnancy. Onset of delivery on term, normal delivery, reduction in abortion, and reduction in any neonatal morbidity and any maternal complication were indicators constituting the domain of maternal and neonatal outcomes.

**Table 1:** Selected indicators to monitor the effectiveness of the interventions on uptake to health care and maternal and neonatal outcomes.

Domain and parameters		Definition
**Uptake of health care services**		
	ANC^a^ visits	Percentage of pregnant women who completed 4 or more ANC visits based on the World Health Organization recommendations
	Supplement intake	Percentage of pregnant women taking the required vitamins that a women should take, including folic acid, iron, calcium, and magnesium
	Ultrasound screenings	Percentage of pregnant women who underwent 2 or more ultrasound screenings during their pregnancy period
	Blood tests	Percentage of pregnant women who underwent the available blood tests at their PHCs^b^, including fasting blood sugar (FBS), blood group (+/–Rh), and the complete blood count (CBC)
	Urine analysis	Percentage of pregnant women who underwent the urine analysis test
**Maternal and neonatal outcomes**		
	Onset of delivery	Percentage of pregnant women who had a term delivery, delivering their latest child after week 37
	Method of delivery	Percentage of pregnant women who had a normal delivery
	Abortion	Percentage of women who had an abortion in their latest pregnancy before week 22
	Neonatal morbidities	Percentage of pregnant women who faced any neonatal complications, including neonatal intensive care unit (NICU) admission, preterm delivery, Iatrogenic preterm birth injury, low birthweight, and neonatal early death
	Maternal complications	Percentage of pregnant women who had any maternal complications, including gestational hypertension, gestational diabetes, perinatal depression, preterm labor, stillbirth, abortion, postpartum infection, postpartum hemorrhage, and postpartum depression

^a^ANC: antenatal care.

^b^PHC: primary health care center.

### Statistical Analysis

Pregnant woman with a nationality other than Lebanese, Syrian, or Palestinian were excluded from the analysis. Sample baseline characteristics were calculated for the intervention and control arms using count and percentages or means (SDs). Chi-square (*χ*^2^) and independent sample *t* tests were conducted to check for all possible differences in the baseline characteristics between the control and intervention arms. Given the fact that all the indicators are categorical, bivariate analysis was performed using the Pearson chi-square (*χ*^2^) test to test the significant difference pre- and postintervention. To evaluate the impact of the intervention, separate logistic regressions were generated for each of the study indicators over the study period in each arm, while controlling for all the possible baseline differences between the control and intervention arms as age, nationality, history of chronic diseases, preterm delivery, abortion, C-section, gravida, and parity. Analysis was carried out at a .05 significance level using Stata SE software.

## Results

### Participants’ Demographic Characteristics

Table 2 presents the baseline demographic and health characteristics of the participants in the control and intervention groups. The number of participants was almost equal between the control (n=1993, 50%) and the intervention (n=1996, 50%) group. At baseline, more than half of the sample was aged between 18 and 29 years in both intervention (801/1996, 54.3%) and control (806/1993, 53%) groups. The majority of the participants were Palestinians, constituting 71.3%(n=1423) of those in the intervention group and 63.5% (n=1267) of those in the control group. The rest of the participants were either Lebanese or Syrians. A higher number of patients in the intervention group (n=160, 8.1%) had a chronic disease. Regarding obstetric history, 6% (n=121) of participants in the intervention group had had a previous preterm delivery, 30.3%(n=605) had had a previous abortion, and 33% (n=664) had had a previous C-section delivery. In contrast, 4.3% (n=85) of participants in the control group had had a preterm delivery, 28.9% (n=575) had had an abortion, and 26.5% (n=529) had had a C-section previously. The mean gravida for the intervention and control groups was 2.98 (SD 2.02) and 3.12 (SD 1.86), respectively. There was a significant difference between the intervention and control groups in all the characteristics except for age.

**Table 2:** Baseline demographic and health characteristics by study group (N=3989).

Characteristics			Intervention group (n=1996)		Control group (n =1993)		*P* value
**Age (years), n (%)**							
	<18	46 (2.3)		48 (2.4)		.50	
	18-29	1083 (54.3)		1057 (53.0)		—^a^	
	30-39	801 (40.1)		806 (40.4)		—	
	≥40	66 (3.3)		82 (4.1)		—	
**Nationality, n (%)**							
	Lebanese	183 (9.2)		228 (11.4)		<.001^b^	
	Palestinian	1423 (71.3)		1267 (63.6)		—	
	Syrian	390 (19.5)		498 (25.0)		—	
**Health and obstetric characteristics**							
	Have chronic diseases, n (%)	160 (8.1)		64 (3.2)		<.001^b^	
	Previous preterm delivery^c^, n (%)	121 (6.1)		85 (4.3)		.01^b^	
	Previous abortion^c^, n (%)	605 (30.3)		575 (28.9)		.15	
	Previous C-section^c,d^, n (%)	664 (33.3)		529 (26.5)		<.001^b^	
	Gravida, mean (SD)	2.98 (2.02)		3.12 (1.86)		<.001^b^	
	Parity, mean (SD)	1.50 (1.34)		1.69 (1.51)		<.001^b^	

^a^Not applicable.

^b^Significant at .05.

^c^Any previous maternal characteristic excludes the latest pregnancy.

^d^C-section: cesarean section.

### Indicators Assessing Uptake of Health Care Services

Results of the bivariate analysis of the selected study indicators are presented in Table 3 across the 2 groups pre- and postintervention. Regarding indicators assessing uptake of health care services, during pregnancy, there was a significant rise in the percentage of women completing 4 or more ANC visits between the pre- and postintervention periods in both intervention and control groups. Specifically, the increase was higher in the intervention group, where the percentage of women completing 4 or more ANC visits increased from 71.9% (n=1436) preintervention to 79.9% (n=1251) postintervention (*P*<.001), while in the control group, the percentage increased from 68.6% (n=1367) preintervention to 74.5% (n=980) postintervention (*P*<.001). A similar trend was noticed in the percentage of women undergoing more than 2 ultrasound screenings, with a significant increase in the percentage from 44.2% (n=881) to 86.1% (n=1323) in the control group and from 46% (n=923) to 80.7% (n=1013) in the intervention group pre- and postintervention, respectively. Likewise, a significant increase was found in the percentage of women who received the intervention and completed lab tests (preintervention: n=1467, 73.5%; postintervention: n=1291, 82.5%; *P*<.001). On the contrary, in the control group, the percentage of women who completed the blood tests available at their corresponding PHCs decreased. Furthermore, there was an overall increase in the percentage of women undergoing urine analysis through their pregnancy, with a higher increase in the intervention group (preintervention: n=1276, 63.9%; postintervention: n=1379, 89.7%; *P*<.001) compared to the control group (preintervention: n=1746, 87.6%; postintervention: n=1161, 94.4%; *P*<.001). Additionally, there was an almost equivalent increase in the percentage of women taking their required supplements pre- and postintervention, respectively, in both intervention (n=906, 45.4%, to n=1162, 74.3%; *P*<.001) and control (n=768, 38.5%, to n=850, 64.6%; *P*<.001) groups.

**Table 3:** Bivariate analysis of the selected outcomes/indicators by study group pre- and postintervention (N=3989).

Indicators			Intervention group (n=1996)							Control group (n=1993)				
			Preintervention		Postintervention		*P* value		Preintervention			Postintervention		*P* value
**Uptake of health care services**														
	ANC^a^ visits (≥4), n (%)	1436 (71.9)		1251 (79.9)		<.001^b^		1367 (68.6)			980 (74.5)		<.001^b^	
	Ultrasound screenings (≥2 images), n (%)	881 (44.2)		1323 (86.1)		<.001^b^		923 (46.3)			1013 (80.7)		<.001^b^	
	Completed lab tests, n (%)	1467 (73.5)		1291 (82.5)		<.001^b^		1556 (78.1)			919 (69.9)		<.001^b^	
	Completed urine analysis, n (%)	1276 (63.9)		1379 (89.7)		<.001^b^		1746 (87.6)			1161 (94.4)		—^c^	
	Took the required supplements, n (%)	906 (45.4)		1162 (74.3)		<.001^b^		768 (38.5)			850 (64.6)		<.001^b^	
**Maternal and neonatal outcomes**														
	Onset of delivery: term, n (%)	1444 (72.5)		1011 (76.9)		.004^b^		1444 (85.4)			1011 (87.6)		.10	
	Neonatal morbidities, n (%)	159 (7.8)		85 (5.4)		.003^b^		69 (3.5)			40 (3.0)		.51	
	Method of delivery: normal, n (%)	962 (51.9)		652 (46.1)		.001^b^		1072 (59.4)			631 (54.0)		.004^b^	
	Abortion, n (%)	143 (7.16)		150 (9.6)		.009^b^		187 (9.4)			146 (11.1)		.11	
	Maternal complications, n (%)	309 (15.5)		407 (26.0)		<.001^b^		311 (15.6)			314 (23.9)		.02^b^	

^a^ANC: antenatal care.

^b^Significant at .05.

^c^Not applicable.

### Maternal and Neonatal Outcomes

Analysis of the data from the charts showed that the onset of delivery improved significantly in the intervention group but not in the control group. After the implementation of the intervention, the proportion of women who had a term delivery increased from 72.5% (n=1444) to 76.9% (n=1011) in the intervention group (*P*=.004). Similar results were observed in regard to neonatal morbidities, where the proportion of women decreased from 7.8% (n=159) to 5.4% (n=85) in the intervention group (*P*=.003). However, the proportion of women who had a normal delivery decreased in both intervention (preintervention: n=962, 51.9%; postintervention: n=652, 46.1%; *P*<.001) and control (preintervention: n=1072, 59.4%; postintervention: n=631, 54%; *P=*.004) groups, with a notable rise in the proportion of woman having C-section deliveries. Results of maternal complications were in the same direction of delivery and abortion, where a significant increase was detected in the proportion of women with maternal complications in both intervention (preintervention: n=309, 15.5%; postintervention: n=407, 26%; *P*<.001) and control (preintervention: n=311, 15.6%; postintervention: n=314, 23.9%; *P*=.02) groups.

### Regression Models

Separate logistic regression models were generated for the control and intervention groups to quantify the intervention’s impact over the study period (pre- and postintervention), while controlling for baseline characteristics that were different across the 2 groups (see Table 2). Each of the 2 models was adjusted for age, nationality, condition of existing chronic diseases, and obstetric history features (ie, previous preterm delivery, previous abortion, previous C-section, gravida, and parity). Tables 4 and 5 show the adjusted odds ratios (ORs) resulting from the logistic regression analysis of the selected indicators. Considering the domain of uptake of health care services, Table 4 indicates that there was a 56.9% increase in the odds of completing more than 4 ANC visits postintervention compared to preintervention (OR 1.569, 95% CI 1.329-1.852, *P*<.001), adjusting for the difference in the demographical, health, and obstetric characteristics. The OR of ANC visits was not statistically significant in the control group (*P*=.58). In contrast, concerning ultrasound images, participants in the intervention group had significantly higher odds (OR 7.984, 95% CI 6.687-9.523, *P*<.001) of having 2 or more images postintervention compared to the control group, where there was also a significant but lower improvement (OR 3.432, 95% CI 2.836-4.153, *P*<.001). A significantly high increase in the odds of pregnant women undergoing urine analysis was observed postintervention in the intervention group (OR 4.399, 95% CI 3.631-5.330, *P*<.001). Concerning lab tests, an 82.1% increase in the odds of pregnant women completing the required blood tests during their pregnancy was noted postintervention compared to preintervention (OR 1.821, 95% CI 1.514-2.191, *P*<.001).

**Table 4:** Adjusted OR^a^ from logistic regression analysis of the selected indicators (study parameters in the uptake of health care services domain) on the study period (pre- and postintervention).

Indicator and study periods			Intervention group (n=1996)			Control group (n=1993)	
		OR (95% CI)		*P* value	OR (95% CI)		*P* value
**ANC^b^ visits (≥4 vs <4 as reference)**							
	Preintervention	—^b^		—	—		—
	Postintervention	1.569^c^ (1.329-1.852)		<.001^d^	1.053^c^ (0.876-1.266)		.58
**Ultrasound images (≥2 vs <2 as reference)**							
	Preintervention	—		—	—		—
	Postintervention	7.984^c^ (6.687-9.532)		<.001^d^	3.432^c^ (2.836-4.152)		<.001^d^
**Supplement uptake (yes vs no as reference)**							
	Preintervention	—		—	—		—
	Postintervention	3.508^c^ (3.020-4.076)		<.001^d^	2.453^c^ (2.055-2.928)		<.001^d^
**Urine analysis (yes vs no as reference)**							
	Preintervention	—		—	—		—
	Postintervention	4.399^c^ (3.631-5.330)		<.001^d^	1.323^c^ (0.966-1.812)		.08
**Lab tests (yes vs no as reference)**							
	Preintervention	—		—	—		—
	Postintervention	1.821^c^ (1.514-2.191)		<.001^d^	0.372^c^ (0.306-0.451)		<.001^d^

^a^OR: odds ratio.

^b^Reference category.

^c^Adjusting for different baseline characteristics: age, nationality, history of chronic diseases, abortion, cesarean section (C-section), gravida, and parity.

^d^Significant at .05.

**Table 5:** Adjusted OR^a^ from logistic regression analysis of the selected indicators (study parameters in the maternal and neonatal outcomes domain) on the study period (pre- and postintervention).

Indicator and study periods			Intervention group (n=1996)			Control group (n=1993)	
		OR (95% CI)		*P* value	OR (95% CI)		*P* value
**Delivery onset (term vs preterm as reference)**							
	Preintervention	—^b^		—	—		—
	Postintervention	1.295^c^ (1.095-1.532)		.002^d^	1.216^c^ (0.944-1.565)		.13
**Delivery type (normal vs C-section as reference)**							
	Preintervention	—		—	—		—
	Postintervention	0.774^c^ (0.657-0.911)		.002^d^	0.865^c^ (0.716-1.046)		.14
**Abortion (no vs yes as reference)**							
	Preintervention	—		—	—		—
	Postintervention	0.702^c^ (0.546-0.902)		.006^d^	0.714^c^ (0.546-0.934)		.01^d^
**Maternal complications (no vs yes as reference)**							
	Preintervention	—		—	—		—
	Postintervention	0.535^c^ (0.449-0.637)		<.001^d^	0.586^c^ (0.474-0.723)		<.001^d^
**Neonatal morbidities (no vs yes as reference)**							
	Preintervention	—		—	—		—
	Postintervention	1.580^c^ (1.185-2.108)		.002^d^	1.353^c^ (0.825-2.218)		.23

^a^OR: odds ratio.

^b^Reference category.

^c^Adjusting for different baseline characteristics: age, nationality, history of chronic diseases, abortion, cesarean section (C-section), gravida, and parity.

^d^Significant at .05.

The analysis of the intervention’s impact on maternal and neonatal outcomes revealed significant findings (Table 5). Specifically, the odds of experiencing a term delivery increased significantly by 29.5% (OR 1.295, 95% CI 1.095-1.532, *P*=.002) only in the intervention group. However, concerning abortion and delivery type, the study period was associated with decreased odds of normal deliveries (OR 0.774, 95% CI 0.657-0.911, *P*=.002) and decreased odds of not having an abortion (OR 0.702, 95% CI 0.657-0.911, *P*=.006). Table 5 also indicates that the odds of not having any maternal complication were 0.535 (95% CI 0.449-0.637, *P*<.001) in the intervention group and 0.586 (95% CI 0.474-0.723, *P*<.001) in the control group. In contrast, there was a significant increase of 58% in the odds of not experiencing any of the neonatal morbidities during the post- intervention period (OR=1.580, 95% CI 1.185-2.108, *P*=.002). Notably, this increase was detected as well in the control group but failed to demonstrate statistical significance.

## Discussion

### Principal Findings

This study assessed the impact of mHealth and AI-based gamified intervention on improving maternal health outcomes and enhancing the uptake of ANC services among disadvantaged Lebanese and refugee pregnant women. The study demonstrated significant improvements in maternal and neonatal health outcomes following the intervention. Pregnant women in the intervention group showed higher odds of completing 4 or more ANC visits (OR 1.569, 95% CI 1.329-1.852), undergoing urine analysis (OR 4.399, 95% CI 3.631-5.330), and completing required blood tests (OR 1.821, 95% CI 1.514-2.191) postintervention. Similarly, the odds of completing 2 or more ultrasound screenings were significantly higher in the intervention group (OR 7.984, 95% CI 6.687-9.523) than in the control group (OR 3.432, 95% CI 2.836-4.153). Supplement uptake also improved significantly in both groups, with a greater increase observed in the intervention group (from 45.4% to 74.3%, *P*<.05). The intervention significantly increased the odds of term delivery (OR 1.295, 95% CI 1.095-1.532) and reduced neonatal morbidities (OR 1.580, 95% CI 1.185-2.108). However, both groups showed decreased odds of a normal delivery and increased maternal complications, highlighting the need for continued efforts to mitigate these outcomes.

### Uptake of Primary Health Care Services

Our study showed that there was an increase in the overall uptake of ANC visits among pregnant women in the intervention group compared to those in the control group. Such findings are in line with other studies demonstrating that implementing mHealth interventions, such as sending maternal health text messages to pregnant women, yields positive outcomes on the uptake of ANC and overall health [76-78]. Recent studies have suggested that sending reminders specifically for health care appointments could be more effective than not sending any reminders at all [79-83]. The increased likelihood of attending at least 4 ANC visits could be explained by the additional maternal health information and psychosocial support provided by the message content generally and the ANC attendance reminder messages specifically in the GAIN MHI intervention. In a fragile context, such as Lebanon, where health care access is limited, such interventions are crucial for improving maternal and neonatal health [22]. Scalable and cost-effective mHealth solutions address systemic barriers in resource-constrained settings, ensuring better care continuity and health outcomes [43].

The higher odds of ultrasound use, completion of laboratory tests, adherence to supplements, and undergoing urinalysis among pregnant women in the intervention group emphasizes the effectiveness of mHealth interventions in promoting and actually improving ANC-seeking behavior. Our findings are consistent with other studies on mHealth and maternal health, where increased uptake of health care services was reported. A study in India demonstrated that the implementation of an mHealth intervention resulted in improved health behaviors among pregnant women, specifically by encouraging consistent adherence to supplementation. Another study in Bangladesh showed that receiving and listening to messages resulted in a significant increase in the rate of completion of laboratory tests [84]. The intervention’s effectiveness might stem from its contextualized approach, which probably helped overcome logistical, linguistic, and geographical barriers, while also increasing awareness among pregnant women. It is important to highlight that substantial progress has been observed in both intervention and control groups, with notably greater improvements seen in the intervention group. This observation may be potentially due to the fact that PHCs in Lebanon benefit from external funding and ongoing projects, under the auspices of the MOPH and the UNRWA, aimed at enhancing overall health and maternal well-being [35-40,85]. The implementation of these projects at the selected PHCs may have contributed to the results obtained. In fragile contexts, such as Lebanon, mHealth interventions play a critical role in overcoming systemic barriers, improving ANC-seeking behaviors, and supporting maternal health outcomes [78]. By addressing logistical and awareness barriers, these interventions improve maternal health outcomes. Notably, progress in both intervention and control groups highlights the role of external funding and initiatives by the MOPH and the UNRWA in strengthening PHC services, further supporting maternal well-being.

### Maternal Outcomes

Our study also showed a significant increase in term deliveries in the intervention group compared to the control group. One possible explanation for this could be that the increased overall uptake of health care services and supplement intake may play a potential role in decreasing the rate of preterm births. Notably, although the increase in the uptake of health care services was evident in both intervention and control groups, the likelihood was greater in the intervention group, possibly due to the encouragement provided by the GAIN MHI mHealth intervention. Additional research conducted by Gillespie [86] and Li et al [87] showed similar findings, suggesting that the rate of preterm births could potentially be decreased through the augmentation of supplement intake.

The increase in the proportion of pregnant women undergoing C-section deliveries in both intervention and control groups without a clear explanation is concerning, especially considering that the unnecessary use of C-sections may lead to negative health consequences and inefficiencies in resource allocation [88-90]. In Lebanon, understanding and addressing this trend is crucial to ensure optimal maternal health outcomes. The rise in C-section rates without medical indications is a worldwide concern and, unfortunately, is expected to persist with nearly a third (29%) of all births likely to take place by C-section by 2030 [91]. This alarming projection emphasizes the urgent need for a concerted effort to address the underlying factors driving this trend. It is essential to recognize that a singular mHealth intervention is insufficient to fully address complex maternal health challenges. This underscores the need for a comprehensive approach involving multiple stakeholders, including HCPs, policymakers, and communities. By prioritizing evidence-based practices and policies, there is an opportunity toward promoting vaginal births, when safe and appropriate, while ensuring that C-sections are reserved for cases where they are medically necessary. This should include providing continuous professional development for HCPs and implementing community education programs to raise awareness about the risks and benefits of different delivery methods. Such measures are essential for safeguarding maternal and neonatal health and reducing the burden of unnecessary surgical interventions.

In both intervention and control groups, there were no observed improvements concerning abortion and maternal complications among the disadvantaged Lebanese and refugee pregnant women. This absence of improvement underscores that maternal health outcomes are intricately influenced by various determinants that extend well beyond clinical interventions, encompassing social, economic, and environmental factors [92,93]. The disadvantaged status of the study population highlights the significant impact of socioeconomic disparities, particularly on women, which not only affect their own well-being but also shape the life prospects of their newborns. Financial constraints, in particular, may contribute to the decision to undergo abortion due to the substantial financial responsibilities associated with raising children. Although the GAIN MHI intervention has yielded promising results, it is evident that merely offering educational messages and appointment reminders is not sufficient when financial barriers and social determinants still hinder the uptake of essential maternal health care services, including addressing and treating maternal complications. Similar results were documented in a study on an mHealth intervention, the MatHealth app, indicating that financial challenges can impede the functionality of the intervention. As such, recognizing the broader determinants of maternal health outcomes, it remains important to address socioeconomic disparities and financial barriers alongside clinical interventions. By engaging in collaborative efforts, including comprehensive support, community empowerment initiatives, and adequate funding mechanisms, equitable improvements in maternal and neonatal health outcomes for disadvantaged populations can be achieved. Persistent socioeconomic barriers, particularly among disadvantaged Lebanese and refugee women, limit the intervention’s impact on maternal complications and abortion rates [22]. Financial constraints and social determinants remain significant obstacles to accessing essential care. Addressing these challenges requires a comprehensive approach that combines mHealth interventions with socioeconomic support, professional development for HCPs, and community education, ensuring equitable maternal and neonatal health outcomes.

### Neonatal Outcomes

In terms of neonatal health outcomes, our study demonstrated a significant increase in the likelihood of not facing any neonatal morbidities postintervention. This aligns with the existing literature, such as the study by Bush et al [94], who found that the implementation of mHealth apps, such as the WYhealth Due Date Plus app, designed for pregnant women, resulted in a marked reduction in the incidence of low birth weight. Similarly, Nishimwe et al [95] observed an improvement in newborn outcomes with the sue of the Safe Delivery mHealth app targeting midwives. These findings underscore the multifaceted approach of the GAIN MHI intervention, which not only prioritizes support for expectant mothers but also extends its impact to their HCPs.

In light of these findings, it is essential to integrate mHealth solutions into the primary health care system, ensuring widespread uptake of maternal health information and appointment reminders. In practice, efforts should focus on contextualizing these interventions to address logistical, linguistic, and geographical barriers and include comprehensive support mechanisms to mitigate socioeconomic disparities, thereby fostering equitable maternal and neonatal health improvements. To effectively address the multifaceted nature of maternal health, it is recommended to integrate mHealth interventions within comprehensive maternal health programs. These programs should address both clinical and nonclinical factors that influence health outcomes, ensuring a holistic approach to maternal care. In fragile contexts, such as Lebanon, improving neonatal health is crucial. The GAIN MHI intervention significantly reduced neonatal morbidities, aligning with studies showing mHealth apps improve birth outcomes by enhancing maternal care and supporting HCPs [78]. Integrating mHealth solutions into primary health care, while addressing logistical and socioeconomic barriers, can foster equitable maternal and neonatal health improvements. A holistic approach that combines mHealth with broader maternal health programs is essential for sustained impact.

### Equity and Gender Considerations

The GAIN MHI project prioritized equity and gender considerations across its design, implementation, and evaluation phases to ensure inclusivity and fairness in addressing maternal health challenges. All eligible women were recruited without discrimination based on race, ethnicity, nationality, religion, or socioeconomic status. The educational interventions were tailored to accommodate varying literacy levels and socioeconomic backgrounds, empowering women to make informed decisions about their health regardless of their educational or financial circumstances.

Recognizing the cultural and social dynamics of the MENA region, the project incorporated a participatory approach that engaged women and their spouses. Weekly educational messages targeted men to foster shared responsibility in maternal health, challenge traditional gender norms, and promote spousal support. This approach addressed critical barriers in underprivileged communities, where women often require the husband’s consent to access health care services or purchase medications.

Additionally, the project leveraged AI and gamification through the GAIN MHI app to enhance the professional development of HCPs. By addressing knowledge gaps and tailoring training to individual needs, the app minimized inequities in access to continuing education for HCPs, particularly those serving disadvantaged populations. The integration of these innovative tools ensured that health care delivery was equitable, culturally appropriate, and responsive to the diverse needs of pregnant women and their communities.

Although the intervention aimed to promote equity, the potential for technology to exacerbate inequities was carefully mitigated. For example, the app was made available in English and Arabic to reduce language barriers, and the inclusion of automated feedback via WhatsApp ensured accessibility for users with limited digital literacy.

### Limitations

Despite the positive outcomes of the GAIN MHI intervention, several limitations and potential biases must be acknowledged. The use of historical controls introduces selection bias, as differences between pre- and postintervention groups may reflect not only the intervention’s impact but also other temporal changes, such as shifts in health care policies, provider practices, or patient demographics. Additionally, reliance on existing medical charts for data collection compromised data quality due to inconsistencies and missing information. Human errors in documentation and incomplete charts at PHCs, often caused by patients seeking care at private clinics or skipping appointments, posed challenges for tracking full medical histories. This fragmented documentation hindered comprehensive data collection and analysis, potentially reducing the reliability of the study’s findings. The exclusive focus on objective data from medical charts also limited the study’s ability to capture qualitative aspects, such as reasons for abortion or barriers to accessing health care, which are critical for understanding patients’ experiences and decision-making processes. This underscores the need for complementary qualitative research to provide a holistic view of the factors influencing health care outcomes.

Cultural norms in the MENA region further complicated the intervention’s implementation. Gender norms may have influenced the women’s and their spouses’ willingness to fully participate, and despite efforts to encourage spousal involvement through educational messages, varying levels of male engagement may have unequally affected maternal health outcomes. The study’s focus on pregnant women attending PHCs also limits the generalizability of its findings to the broader population, as women facing logistical, financial, or social barriers to accessing care were likely excluded, introducing another layer of selection bias.

Lastly, although the intervention aimed to reduce inequities by targeting underprivileged populations and incorporating AI-based tools for HCP training, reliance on technology may have inadvertently introduced disparities. Variations in access to smartphones or digital literacy levels among participants could have affected the intervention’s reach and impact. These limitations highlight the need to interpret the findings with caution and emphasize the importance of further research to validate and refine the intervention for broader and more equitable.

### Conclusion

In conclusion, this study highlights the effectiveness of the AI-based gamified mHealth intervention GAIN MHI in enhancing the uptake of ANC and improving maternal and neonatal outcomes. Although encouraging results were observed, the study highlights the need to couple the implementation of such interventions with other complementary interventions targeted toward addressing socioeconomic and context-specific factors that may contribute to the achievement of improved maternal and neonatal health outcomes among disadvantaged populations.

## Abbreviations

AI: artificial Intelligence
ANC: antenatal care
C-section: cesarean section
EU: European Union
GAIN MHI: Gamification, Artificial Intelligence and mHealth Network for Maternal Health Improvement
HCP: health care provider
IRB: Institutional Review Board
KAP: knowledge, attitude, and practices
MENA: Middle East and North Africa
mHealth: mobile health
MOPH: Ministry of Public Health
NGO: nongovernmental organization
OBGYN: obstetricians and gynecologists
OR: odds ratio
PHC: primary health care center
PNC: postnatal care
UNRWA: United Nations Relief and Works Agency for Palestine Refugees in the Near East

## References

[1] Benage M, Greenough PG, Vinck P, Omeira N, Pham P (2015). An assessment of antenatal care among Syrian refugees in Lebanon. Confl Health.

[2] Iyakaremye I, Mukagatare C (2016). Forced migration and sexual abuse: experience of Congolese adolescent girls in Kigeme refugee camp, Rwanda. Health Psychol Rep.

[3] Samari G (2017). Syrian refugee women's health in Lebanon, Turkey, and Jordan and recommendations for improved practice. World Med Health Policy.

[4] Singh N, Smith J, Khosla R, Say L, Blanchet K, Aryasinghe (2018). A long way to go: a systematic review to assess the utilisation of sexual and reproductive health services during humanitarian crises. BMJ Glob Health.

[5] Makhoul G, Falakha G, Makhoul CN, AbdelAhad A (2015). International perspectives: impact of Syrian refugees on neonatal care in Hopital Notre Dame de la Paix, Akkar, North Lebanon. NeoReviews.

[6] Nabulsi D, Abou Saad M, Ismail H, Doumit MAA, El-Jamil F, Kobeissi L, Fouad FM (2021). Minimum initial service package (MISP) for sexual and reproductive health for women in a displacement setting: a narrative review on the Syrian refugee crisis in Lebanon. Reprod Health.

[7] Usta J, Masterson AR, Djamba YK, Kimuna SR (2015). Women and health in refugee settings: the case of displaced Syrian women in Lebanon. Gender-Based Violence: Perspectives from Africa, the Middle East, and India.

[8] Moolla A, Mdewa W, Erzse A, Hofman K, Thsehla E, Goldstein S, Kohli-Lynch C (2024). A cost-effectiveness analysis of a South African pregnancy support grant. PLOS Glob Public Health.

[9] Raatikainen K, Heiskanen N, Heinonen S (2007). Under-attending free antenatal care is associated with adverse pregnancy outcomes. BMC Public Health.

[10] (2007). Standards for maternal and neonatal care. World Health Organization.

[11] Essaid A, Shukri S, El Gharaibeh Y, Abu Taleb H, Awwad N, Nour H, Usta J, Clark CJ, Spencer R (2015). Gender Based Violence against Women and Girls Displaced by the Syrian Conflict in South Lebanon and North Jordan: Scope of Violence and Health Correlates.

[12] Huster KMJ, Patterson N, Schilperoord M, Spiegel P (2014). Cesarean sections among Syrian refugees in Lebanon from December 2012/January 2013 to June 2013: probable causes and recommendations. Yale J Biol Med.

[13] Abdin L (2018). Challenges for pregnant Syrian refugees in Lebanon. East Mediterr Health J.

[14] Mourtada R, Schlecht J, DeJong J (2017). A qualitative study exploring child marriage practices among Syrian conflict-affected populations in Lebanon. Confl Health.

[15] (2013). Health UNHCR monthly update Lebanon. UNHCR.

[16] Rizkianti A, Saptarini I, Rachmalina R (2021). Perceived barriers in accessing health care and the risk of pregnancy complications in Indonesia. IJWH.

[17] Gogoi M, Unisa S, Prusty RK (2014). Utilization of maternal health care services and reproductive health complications in Assam, India. J Public Health.

[18] (2023). Fact sheet. Lebanon. UNHCR.

[19] Knudsen AJ, Di Peri R, Meier D (2017). Syria’s refugees in Lebanon: brothers, burden, and bone of contention. Lebanon Facing the Arab Uprisings: Constraints and Adaptation.

[20] (2023). Where we work. UNRWA.

[21] Khattar G, Hallit J, El Chamieh C, Bou Sanayeh E (2022). Cardiovascular drug shortages in Lebanon: a broken heart. Health Econ Rev.

[22] Bou Sanayeh E, El Chamieh C (2023). The fragile healthcare system in Lebanon: sounding the alarm about its possible collapse. Health Econ Rev.

[23] Dumit N, Honein-AbouHaidar G (2019). The impact of the Syrian refugee crisis on nurses and the healthcare system in Lebanon: a qualitative exploratory study. J Nurs Scholarsh.

[24] Devi S (2020). Economic crisis hits Lebanese health care. Lancet.

[25] Nemr E, Moussallem M, Nemr R, Kosremelli Asmar M (2023). Exodus of Lebanese doctors in times of crisis: a qualitative study. Front Health Serv.

[26] Bou Sanayeh E, El Chamieh C, Saade MC, Maalouf RG, Bizri M (2022). Post-traumatic stress symptoms experienced by healthcare workers in Lebanon four months following Beirut's ammonium nitrate explosion: a survey-based study. Arch Public Health.

[27] Daigle M, Spencer A, Diab JL, Samneh B, Afandi A (2023). Sex, health and rights in displacement and humanitarian response: crises upon crises in Lebanon and beyond. ODI Global.

[28] AlArab N, Nabulsi D, El Arnaout N, Dimassi H, Harb R, Lahoud J, Nahouli L, Abou Koura A, El Saddik G, Saleh S (2023). Reproductive health of Syrian refugee women in Lebanon: a descriptive analysis of the Sijilli electronic health records database. BMC Womens Health.

[29] El Arnaout N, Rutherford S, Zreik T, Nabulsi D, Yassin N, Saleh S (2019). Assessment of the health needs of Syrian refugees in Lebanon and Syria's neighboring countries. Confl Health.

[30] Health in Lebanon. UNRWA.

[31] Hemadeh R, Hammoud R, Kdouh O (2019). Lebanon's essential health care benefit package: a gateway for universal health coverage. Int J Health Plann Manag.

[32] The Ministry of Public Health. Ministry of Public Health (MOPH).

[33] MOPH (2018). Primary Health Care Department annual dashboard - 2018. Ministry of Public Health (MOPH).

[34] Parkinson S, Behrouzan O (2015). Negotiating health and life: Syrian refugees and the politics of access in Lebanon. Soc Sci Med.

[35] Abdulrahim S, El Rafei R, Beydoun Z, El Hayek GY, Nakad P, Yunis K (2019). A test of the epidemiological paradox in a context of forced migration: low birthweight among Syrian newborns in Lebanon. Int J Epidemiol.

[36] (2024). Reproductive health services. Ministry of Public Health (MOPH).

[37] (2022). Department of Internal Oversight Services 2022 annual report. UNRWA.

[38] EU/IfS project. Conflict reduction through improving healthcare services for the vulnerable population in Lebanon. Ministry of Public Health (MOPH).

[39] (2024). Lebanon Health Resilience Project (P163476): second semi-annual progress report. Ministry of Public Health (MOPH).

[40] (2018). EU biggest donor to the Lebanese health sector. European Union.

[41] (2024). Our vision/mission. Ministry of Public Health (MOPH).

[42] (2023). EU and UNFPA step up support for vulnerable women in Lebanon during crisis. UNFPA.

[43] Rahman M, Yamaji N, Nagamatsu Y, Ota E (2022). Effects of mHealth interventions on improving antenatal care visits and skilled delivery care in low- and middle-income countries: systematic review and meta-analysis. J Med Internet Res.

[44] Fedha T (2014). Impact of mobile telephone on maternal health service care: a case of Njoro division. Open J Prev Med.

[45] Higgs E, Goldberg AB, Labrique AB, Cook SH, Schmid C, Cole CF, Obregón RA (2014). Understanding the role of mHealth and other media interventions for behavior change to enhance child survival and development in low- and middle-income countries: an evidence review. J Health Commun.

[46] Miah S, Gammack J, Hasan N (2017). Extending the framework for mobile health information systems research: a content analysis. Inf Syst.

[47] Okolo CA, Babawarun O, Olorunsogo TO (2024). Mobile health (mhealth) innovations for public health feedback: a global perspective. Int Med Sci Res J.

[48] De P, Pradhan MR (2023). Effectiveness of mobile technology and utilization of maternal and neonatal healthcare in low and middle-income countries (LMICs): a systematic review. BMC Womens Health.

[49] WHO Team (2023). Trends in maternal mortality 2000 to 2020: estimates by WHO, UNICEF, UNFPA, World Bank Group and UNDESA/Population Division. World Health Organization.

[50] Barron P, Peter J, LeFevre AE, Sebidi J, Bekker M, Allen R, Parsons AN, Benjamin P, Pillay Y (2018). Mobile health messaging service and helpdesk for South African mothers (MomConnect): history, successes and challenges. BMJ Glob Health.

[51] Lebrun V, Dulli L, Alami SO, Sidiqi A, Sultani AS, Rastagar SH, Halimzai I, Ahmadzai S, Todd CS (2020). Feasibility and acceptability of an adapted mobile phone message program and changes in maternal and newborn health knowledge in four provinces of Afghanistan: single-group pre-post assessment study. JMIR Mhealth Uhealth.

[52] Maliwichi P, Chigona W, Sowon K (2021). Appropriation of mHealth interventions for maternal health care in sub-Saharan Africa: hermeneutic review. JMIR Mhealth Uhealth.

[53] Shaheen MY Applications of artificial intelligence (AI) in healthcare: a review. ScienceOpen. Preprint posted online 2021. [doi:10.14293/S2199-1006.1.SOR-.PPVRY8K.v1].

[54] Saif-Ur-Rahman K, Islam MS, Alaboson J, Ola O, Hasan I, Islam N, Mainali S, Martina T, Silenga E, Muyangana M, Joarder T (2023). Artificial intelligence and digital health in improving primary health care service delivery in LMICs: a systematic review. J Evid Based Med.

[55] Wahl B, Cossy-Gantner A, Germann S, Schwalbe NR (2018). Artificial intelligence (AI) and global health: how can AI contribute to health in resource-poor settings?. BMJ Glob Health.

[56] Khan M, Khurshid M, Vatsa M, Singh R, Duggal M, Singh K (2022). On AI approaches for promoting maternal and neonatal health in low resource settings: a review. Front Public Health.

[57] Rittenhouse K, Vwalika B, Keil A, Winston J, Stoner M, Price JT, Kapasa M, Mubambe M, Banda V, Muunga W, Stringer JSA (2019). Improving preterm newborn identification in low-resource settings with machine learning. PLoS One.

[58] Panda PK, Sharma R (2024). Transforming maternal healthcare: harnessing the power of artificial intelligence for improved outcomes and access. World J Adv Res Rev.

[59] Orjuela MAV, Uribe-Quevedo A, Jaimes N, Perez-Gutierrez B (2015). External automatic defibrillator game-based learning app.

[60] Guana V, Xiang T, Zhang H, Schepens E, Stroulia E (2014). UnderControl an educational serious-game for reproductive health.

[61] Molnar A, Kostkova P (2018). Learning about hygiene and antibiotic resistance through mobile games: evaluation of learning effectiveness. Kostkova, Learning about Hygiene and Antibiotic Resistance through Mobile Gamesvaluation of Learning Effectiveness, in Proceedings of the 2018 International Conference on Digital Health.

[62] Miller A, Cafazzo JA, Seto E (2016). A game plan: gamification design principles in mHealth applications for chronic disease management. Health Inform J.

[63] Kayastha R, Mueller S, Yadav P, Kelman I, Boscor A, Saville N, Arjyal A, Baral S, Fordham M, Hearn G, Kostkova P (2021). Do women in Nepal like playing a mobile game? MANTRA: a mobile gamified app for improving healthcare seeking behavior in rural Nepal. Front Public Health.

[64] Talhouk R, Akik C, Araujo-Soares V, Ahmad B, Mesmar S, Olivier P, Balaam M, Montague K, Garbett A, Ghattas H (2020). Integrating health technologies in health services for Syrian refugees in Lebanon: qualitative study. J Med Internet Res.

[65] Yassin N, Khodor R, Baroud M (2018). m-Health for healthcare delivery reform: prospects for Lebanese and refugee communities. Healthcare Systems, 1st Edition.

[66] Braithwaite J, Mannion R, Matsuyama Y, Shekelle PG, Whittaker S, Al-Adawi S, Ludlow K, James W, Ting HP, Herkes J, McPherson E, Churruca K, Lamprell G, Ellis LA, Boyling C, Warwick M, Pomare C, Nicklin W, Hughes CF (2018). The future of health systems to 2030: a roadmap for global progress and sustainability. Int J Qual Health Care.

[67] Doocy S, Paik KE, Lyles E, Hei Tam H, Fahed Z, Winkler E, Kontunen K, Mkanna A, Burnham G (2017). Guidelines and mHealth to improve quality of hypertension and type 2 diabetes care for vulnerable populations in Lebanon: longitudinal cohort study. JMIR Mhealth Uhealth.

[68] Saleh S, Farah A, Dimassi H, El Arnaout N, Constantin J, Osman M, El Morr C, Alameddine M (2018). Using mobile health to enhance outcomes of noncommunicable diseases care in rural settings and refugee camps: randomized controlled trial. JMIR Mhealth Uhealth.

[69] Lakkis N, Atfeh AMA, El-Zein YR, Mahmassani DM, Hamadeh GN (2011). The effect of two types of SMS-texts on the uptake of screening mammogram: a randomized controlled trial. Prev Med.

[70] Bardus M, Ali A, Demachkieh F, Hamadeh G (2019). Assessing the quality of mobile phone apps for weight management: user-centered study with employees from a Lebanese university. JMIR Mhealth Uhealth.

[71] Seita A, Ballout G, Albeik S, Salameh Z, Zeidan W, Shah S, Atallah S, Horino M (2024). Leveraging digital health data to transform the United Nations Systems for Palestine Refugees for the post pandemic time. Health Syst Reform.

[72] Alsswey A, Al-Samarraie H, Bervell B (2021). mHealth technology utilization in the Arab world : a systematic review of systems, usage, and challenges. Health Technol.

[73] Qasrawi R, Amro M, VicunaPolo S, Abu Al-Halawa D, Agha H, Abu Seir R, Hoteit M, Hoteit R, Allehdan S, Behzad N, Bookari K, AlKhalaf M, Al-Sabbah H, Badran E, Tayyem R (2022). Machine learning techniques for predicting depression and anxiety in pregnant and postpartum women during the COVID-19 pandemic: a cross-sectional regional study. F1000Res.

[74] El-Jardali F, Alameddine M, Jamal D, Dimassi H, Dumit NY, McEwen MK, Jaafar M, Murray SF (2013). A national study on nurses' retention in healthcare facilities in underserved areas in Lebanon. Hum Resour Health.

[75] Jaana M, Majdalani M, Tamim H, Rahbany R (2018). Perceived healthcare workforce needs in Lebanon: a step towards informed human resources planning and professional development. East Mediterr Health J.

[76] Endehabtu B, Weldeab A, Were M, Lester R, Worku A, Tilahun B (2018). Mobile phone access and willingness among mothers to receive a text-based mHealth intervention to improve prenatal care in northwest Ethiopia: cross-sectional study. JMIR Pediatr Parent.

[77] Murthy N, Chandrasekharan S, Prakash MP, Ganju A, Peter J, Kaonga N, Mechael P (2020). Effects of an mHealth voice message service (mMitra) on maternal health knowledge and practices of low-income women in India: findings from a pseudo-randomized controlled trial. BMC Public Health.

[78] Bossman E, Johansen MA, Zanaboni P (2022). mHealth interventions to reduce maternal and child mortality in sub-Saharan Africa and southern Asia: a systematic literature review. Front Glob Womens Health.

[79] Free C, Phillips G, Galli L, Watson L, Felix L, Edwards P, Patel V, Haines A (2013). The effectiveness of mobile-health technology-based health behaviour change or disease management interventions for health care consumers: a systematic review. PLoS Med.

[80] Gurol‐Urganci I (2013). Mobile phone messaging reminders for attendance at healthcare appointments. Cochrane Database Syst Rev.

[81] Guy R, Hocking J, Wand H, Stott S, Ali H, Kaldor J (2012). How effective are short message service reminders at increasing clinic attendance? A meta-analysis and systematic review. Health Serv Res.

[82] Alam M, D'Este C, Banwell C, Lokuge K (2017). The impact of mobile phone based messages on maternal and child healthcare behaviour: a retrospective cross-sectional survey in Bangladesh. BMC Health Serv Res.

[83] Opon S, Tenambergen WM, Njoroge KM (2020). The effect of patient reminders in reducing missed appointment in medical settings: a systematic review. PAMJ-OH.

[84] Chowdhury M, Shiblee SI, Jones HE (2019). Does mHealth voice messaging work for improving knowledge and practice of maternal and newborn healthcare?. BMC Med Inform Decis Mak.

[85] (2018). New report: Decline of UNRWA services, consequences will be catastrophic. Euro-Med Human Rights Monitor.

[86] Gillespie B, Anumba DOC, Jayasooriya SM (2022). Nutritional status and the risk of preterm birth. Evidence Based Global Health Manual for Preterm Birth Risk Assessment.

[87] Li B, Zhang X, Peng X, Zhang S, Wang X, Zhu C (2019). Folic acid and risk of preterm birth: a meta-analysis. Front Neurosci.

[88] Keag O, Norman JE, Stock SJ (2018). Long-term risks and benefits associated with cesarean delivery for mother, baby, and subsequent pregnancies: systematic review and meta-analysis. PLoS Med.

[89] Sandall J, Tribe RM, Avery L, Mola G, Visser GH, Homer CS, Gibbons D, Kelly NM, Kennedy HP, Kidanto H, Taylor P, Temmerman M (2018). Short-term and long-term effects of caesarean section on the health of women and children. Lancet.

[90] Sobhy S, Arroyo-Manzano D, Murugesu N, Karthikeyan G, Kumar V, Kaur I, Fernandez E, Gundabattula Sr, Betran Ap, Khan K, Zamora J, Thangaratinam S (2019). Maternal and perinatal mortality and complications associated with caesarean section in low-income and middle-income countries: a systematic review and meta-analysis. Lancet.

[91] (2021). Caesarean section rates continue to rise, amid growing inequalities in access. World Health Organization.

[92] Janaki JS, Soubramanian P (2024). Examining socioeconomic factors influencing maternal health in pregnancy. J Hum Behav Soc Environ.

[93] Thomson K, Moffat M, Arisa O, Jesurasa A, Richmond C, Odeniyi A, Bambra C, Rankin J, Brown H, Bishop J, Wing S, McNaughton A, Heslehurst N (2021). Socioeconomic inequalities and adverse pregnancy outcomes in the UK and Republic of Ireland: a systematic review and meta-analysis. BMJ Open.

[94] Bush J, Barlow DE, Echols J, Wilkerson J, Bellevin K (2017). Impact of a mobile health application on user engagement and pregnancy outcomes among Wyoming Medicaid members. Telemed J E Health.

[95] Nishimwe A, Ibisomi L, Nyssen M, Conco DN (2022). The effect of a decision-support mHealth application on maternal and neonatal outcomes in two district hospitals in Rwanda: pre - post intervention study. BMC Pregnancy Childbirth.

